# The Application of Cinnamon Twig Extract as an Inhibitor of Listeriolysin O against *Listeria monocytogenes* Infection

**DOI:** 10.3390/molecules28041625

**Published:** 2023-02-08

**Authors:** Xiaoning Hou, Qiushuang Sheng, Jichuan Zhang, Runbao Du, Nan Wang, Haoyu Zhu, Xuming Deng, Zhongmei Wen, Jianfeng Wang, Yonglin Zhou, Dan Li

**Affiliations:** 1 Center for Pathogen Biology and Infectious Diseases, Key Laboratory of Organ Regeneration and Transplantation of the Ministry of Education, Department of Respiratory Medicine, The First Hospital of Jilin University, Changchun 130021, China; 2 State Key Laboratory for Zoonotic Diseases, Key Laboratory of Zoonosis Research, Ministry of Education, Institute of Zoonosis, College of Veterinary Medicine, Jilin University, Changchun 130012, China; 3 Key Laboratory for Molecular Enzymology and Engineering of Ministry of Education, School of Life Sciences, Jilin University, Changchun 130012, China

**Keywords:** *Listeria monocytogenes*, listeriolysin O, inhibitor, cinnamon twig extract, anti-virulence, infection, oligomerization

## Abstract

As a major virulence factor of *Listeria monocytogenes* (*L. monocytogenes*), listeriolysin O (LLO) can assist in the immune escape of *L. monocytogenes*, which is critical for the pathogen to evade host immune recognition, leading to various infectious diseases. Cinnamon twig (CT), as a traditional medicine, has been widely used in clinics for multiple functions and it has exhibited excellent safety, efficacy and stability. There are few reports on the effects of the extracts of traditional medicine on bacterial virulence factors. CT has not been reported to be effective in the treatment of *L. monocytogenes* infection. Therefore, this study aims to explore the preventive effect of CT against *L. monocytogenes* infection in vivo and in vitro by targeting LLO. Firstly, a hemolysis assay and a cell viability determination are used to detect the effect of CT extract on the inhibition of the cytolytic activity of LLO. The potential mechanism through which CT extract inhibits LLO activity is predicted through network pharmacology, molecular docking assay, real-time polymerase chain reaction (RT-PCR), Western blotting and circular dichroism (CD) analysis. The experimental therapeutic effect of CT extract is examined in a mouse model infected with *L. monocytogenes*. Then, the ingredients are identified through a high-performance liquid chromatography (HPLC) and thin layer chromatography (TLC) analysis. Here we find that CT extract, containing mainly cinnamic acid, cinnamaldehyde, β-sitosterol, taxifolin, catechin and epicatechin, shows a potential inhibition of LLO-mediated hemolysis without any antimicrobial activity. The results of the mechanism research show that CT extract treatment can simultaneously inhibit LLO expression and oligomerization. Furthermore, the addition of CT extract led to a remarkable alleviation of LLO-induced cytotoxicity. After treatment with CT extract, the mortality, bacterial load, pathological damage and inflammatory responses of infected mice are significantly reduced when compared with the untreated group. This study suggests that CT extract can be a novel and multicomponent inhibitor of LLO with multiple strategies against *L. monocytogenes* infection, which could be further developed into a novel treatment for infections caused by *L. monocytogenes*.

## 1. Introduction

*Listeria monocytogenes (L. monocytogenes),* as a food-borne pathogen, threatens human health and public safety, especially for the elderly, pregnant women and newborns. *L. monocytogenes* is also an important opportunistic pathogen for zoonosis, leading to serious economic losses in animal husbandry [1]. *L. monocytogenes* has been listed by the World Health Organization as one of the four major foodborne pathogenic bacteria (including *L. monocytogenes*, *Escherichia coli*, *Salmonella* and *Staphylococcus aureus*) and has become one of the top three bacteria (the other two are *Toxoplasma gondii* and *Salmonella*) threatening life by serious infectious diseases currently [2]. The incidence of *L. monocytogenes* infection was 2 to 15 cases per 100,000 population with a mortality as high as 30% in developed countries [3]. Since 1985, the Foodborne Disease Outbreak Surveillance System in the United States recorded two mass poisoning incidents involving *L. monocytogenes* infection, resulting in nearly 100 people dead [4]. Therefore, there is an urgent need to monitor and control *L. monocytogenes* infection as even though *L. monocytogenes* is a rare microbe, it is deadly [5]. Antibiotic therapy is the primary strategy for the treatment of *L. monocytogenes* infections under clinical conditions. However, the emergence of multiple antibiotic-resistant *L. monocytogenes* has triggered a growing global crisis in recent years. *L. monocytogenes* has been reported to be highly resistant to tetracycline, cephalosporin antibiotics, ciprofloxacin and erythromycin [6,7,8]. Therefore, there is an urgent need to tackle the antibiotic resistance of *L. monocytogenes* with new therapeutic approaches. Antivirulence strategies that have demonstrated efficacy by reducing the pathogenicity of the pathogen may be complementary to conventional antibiotic therapy when resistance emerges.

Notably, the multiple virulence factors of *L. monocytogenes* play a crucial role in the bacterial disease process. With the emergence and increasing severity of bacterial resistance, there is an urgent need for alternative anti-infective strategies to address this crisis. In addition to traditional antibiotic therapy, targeting bacterial virulence factors has been shown to be an alternative therapeutic strategy for infectious diseases as it puts tremendous pressure on the selection of causative organisms and is detrimental to the development of drug resistance. *L. monocytogenes* is an intracellular bacterium with three pathogenicity islands, LIPI-1, LIPI-2 and LIPI-3 in the genome, which co-regulate the expression of a series of virulence factors, similar to internalin, listeriolysin O, phospholipase C and actin aggregation protein (ActA) [3]. At the beginning of an *L. monocytogenes* infection, the virulence factors are expressed at different stages of its intracellular invasion process and play a vital role in assisting the pathogenicity of *L. monocytogenes* [9,10]. Among these virulence factors, LIPI-1 regulates the expression of LLO, as a member of the cholesterol-dependent cytolysin family, which is the key factor mediating the internalization of *L. monocytogenes* and its escape from phagocytic vacuoles into the cytoplasm. LLO is a cholesterol-dependent cytolysin (CDC) that binds to host cell membranes in a cholesterol-dependent manner [11]. Then, LLO monomers oligomerize into large ring prepore assemblies containing 30–50 subunits, which form a β-barrel pore of approximately 50 nm in diameter that spans the lipid bilayer of various host cells [12]. Briefly, LLO, as an aqueous monomer, forms the oligomer into a ring via a conformational change to form the membrane-damaging pores, and then intracellular Ca^2+^ increases drastically, leading to cell lysis. The vacuole was destroyed by LLO to help *L. monocytogenes* escape successfully. LLO-deficient *L. monocytogenes*, once trapped by the host cell, have been confirmed to demonstrate a poor ability for cellular internalization and proliferation [13]. Consistently, LLO knockout *L. monocytogenes*-infected mice had significantly lower mortality rates compared to wild-type mice [14]. Importantly, extracellular LLO can also penetrate the host cell membrane and cause *L. monocytogenes* infection by disrupting ionic homeostasis and triggering an inflammatory response. Notably, the alteration of Ca^2+^ homeostasis subsequent to LLO pore formation would favor infection via remodeling of the mitochondrial network and endoplasmic reticulum (ER) response [15,16]. Moreover, extracellular LLO has been reported to be sufficient for the induction of *L. monocytogenes* internalization into some epithelial cell lines by perforating the plasma membrane [17]. Recently, it has been reported that the host cell signaling pathway activated by extracellular LLO pore formation can significantly affect the subsequent vacuole escape of *L. monocytogenes*, which may be related to the influence of ion homeostasis on the endosomal network [18]. The multifaceted activity of LLO renders it a reliable target for developable therapies against *L. monocytogenes* infection. Thus, finding a treatment for this virulence factor is one of the most important ways to treat *L. monocytogenes* infections.

As a previous study showed, some natural compounds have been screened as inhibitors of LLO against the *L. monocytogenes* infection [19]. Thus, some studies have reported that natural compounds (e.g., curcumin, epigallocatechin gallate, gallate and fisetin) could effectively inhibit the hemolysis of LLO [20,21,22,23,24,25,26]. Among the abundance of natural compounds, a multicomponent inhibitor extracted from the leaves and branches of cinnamon exhibits a wide range of pharmacological characteristics, including antioxidant, anti-inflammatory and hypoglycemic properties [27,28]. Additionally, CT extract has been used in multiherbal preparations, such as *Ramuli Cinnamomi* and glycyrrhizae decoction, which have proven to be effective therapeutic prescriptions for the treatment of arrhythmia [29]. To date, the potential effects of CT extract on *L. monocytogenes* infection have not been explored. The medicinal ingredients of *Cinnamomum cassia* include *Osmanthus fragrans Lour* (OFL), CT, *Cinnamomi cortex* (CC) and *Bark of Japanese Cinnamon* (BJC), all of which have different pharmacological effects [30]. *Cinnamomum cassia* and its active ingredients have great potential as a traditional Chinese herb against *L. monocytogenes* infection with anti-infectious properties. Herein, we screened the effective medicinal parts of cinnamon and targeted LLO to find an effective means to resist *L. monocytogenes* infection.

In this study, we found that a plant extract obtained from the branches of *Cinnamomum cassia* was a potential LLO inhibitor of *L. monocytogenes* infection. Further study revealed that CT extract treatment simultaneously inhibited LLO expression and oligomerization. The addition of CT extract led to a remarkable alleviation of LLO-induced cytotoxicity. In the in vivo study, after treatment with CT extract, the mortality, bacterial load, pathological damage and inflammatory responses of infected mice were significantly reduced compared with the untreated group. The network pharmacology and molecular docking approaches were used to predict the mechanism of the CT extract for the treatment of *L. monocytogenes* infection. These results provide a new strategy for the rapid development of this natural herbal extract as an agent against bacterial infection.

## 2. Results and Discussion

### 2.1. Hemolysis Inhibition Assay

**Figure 1:**
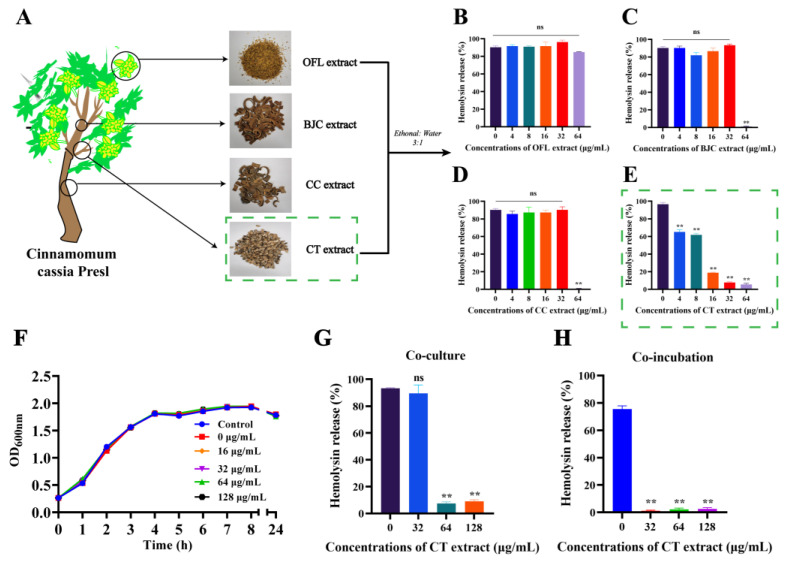
The effect of active components of Cinnamomum cassia on LLO activity. (**A**) The four extractions (OFL extract, BJC extract, CC extract and CT extract) from different parts of *Cortex Cinnamomi*. The hemolysis of purified LLO pre-treated with the indicated concentrations of OFL extract (**B**), BJC extract (**C**), CC extract (**D**) or CT extract (**E**) was determined using a hemolysis assay. (**F**) Growth curve of *L. monocytogenes* co-cultured with different concentrations of CT extract. (**G**) Hemolysis of the culture supernatants from *L. monocytogenes* co-cultured with CT extract. (**H**) Inhibition of hemolysis of *L. monocytogenes* supernatants pre-treated with the indicated concentrations of CT extract. The samples for hemolysis assay treated with PBS were regarded as a negative control and those treated with 0.2% Triton-X 100 were regarded as a positive control (100% hemolysis). ** *p* < 0.01 compared to the samples without extraction treatment. ns, not significant.

The medicinal ingredients of *Cinnamomum cassia*, such as young shoots (cinnamon sticks), young fruits (cinnamon cloves) and leaves, have various types of biological activity against antioxidant, anti-inflammatory, hypoglycemic and anticardiovascular diseases. Herein, Cinnamomum cassia extracts of OFL extract, BJC extract, CC extract and CT extract were used to determine the inhibitory effect on LLO-mediated hemolysis (Figure 1A). Notably, CT extract inhibition was the most significant at a concentration of 4 μg/mL, with the hemolysis of LLO decreasing from 96.60% to 65.12%, and decreasing to 7.7% at a concentration of 32 μg/mL. OFL, CC and BJC extracts did not show significant inhibition of LLO-mediated hemolysis activity at concentrations of 4-32 μg/mL. CT extract can inhibit LLO at much lower concentrations. The difference in the inhibitory effects of CT, BJC and CC extract on LLO was not significant at concentrations of 64 μg/mL and the difference in hemolysis release was within 4%. Thus, we chose CT extract over the other three extracts for the follow-up experiments (Figure 1B–E). CT extract did not influence the growth of L. monocytogenes at the concentrations that showed significant inhibitory activity against LLO, and it showed no antibacterial activity against *L.monocytogenes* EGD strains at a concentration of 128 μg/mL (Figure 1F). In addition, the same inhibitory effect of CT extract on LLO-mediated hemolysis was also observed when co-cultured with *L. monocytogenes* or co-incubated (Figure 1G) with *L. monocytogenes* supernatant (Figure 1H). These results indicate that CT extract can effectively inhibit the hemolysis of LLO without inhibiting the growth of bacteria.

### 2.2. Cell Protection Experiments

**Figure 2:**
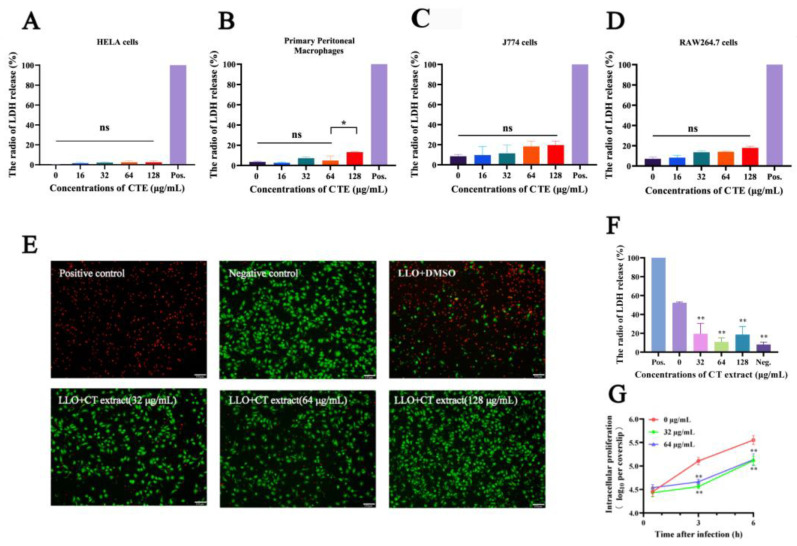
CT extract decreased *L. monocytogenes*-induced cellular injury in a dose-dependent manner. Cytotoxicity of CT extract on HeLa cells (**A**), the primary periodontal macrophages (**B**), J774 cells (**C**) and RAW264.7 cells (**D**). Cytotoxicity was determined by relative lactate dehydrogenase (LDH) release following a 6 h incubation with the indicated concentrations of CT extract. Triton-X 100 at a concentration of 0.2% served as a positive control and no treatment as a negative control. (**E**) The viability of *L. monocytogenes*-infected RAW264.7 cells was assessed with a LIVE/DEAD^®^ kit following the indicated treatment. Then cells co-cultured with LLO + DMSO and 32 μg/mL, 64 μg/mL and 128 μg/mL of CT extract were used to assess cell viability. Triton-X 100 at a concentration of 0.2% served as a positive control and no treatment as a negative control. These images were collected from an original magnification of 40 × (the scale length is 100 μm) (**F**) Cell cytotoxicity induced by LLO in the presence of the indicated concentrations of CT extract was measured by LDH release assay. Cells treated with 0.2% Triton-X 100 or no treatment served as the positive control and negative control, respectively. (**G**) Inhibition of intracellular bacterial growth by CT extract (32 and 64 μg/mL). RAW264.7 macrophages were infected with overnight-cultured *L. monocytogenes* EGD at an MOI of 10. The macrophages were lysed at the indicated time points to determine the numbers of total CFU. * *p* < 0.05 and ** *p* < 0.01 compared to the positive group. ns, not significant.

LLO-mediated cytotoxicity can directly lead to the death of a variety of cells, such as erythrocytes and macrophages [19]. The cytotoxicity of CT extract was preliminarily assessed using LDH assays. The results showed that CT extract hardly exhibited cytotoxicity with different sources of cells such as HeLa cells, primary peritoneal macrophage, J774 cells and RAW264.7 cells at concentrations less than 128 µg/mL for 6 h (Figure 2A–D). Compared with the Triton-X 100 treatment group, cell mortality in CT extract-treated group was lower than 20% when the concentrations were below 128 µg/mL. No significant differences were observed when comparing to the negative control group. As an extracellular pore-forming toxin, LLO also perforated the host cell membrane and thus caused appreciable cell death independent of host cell invasion [31]. The visual images of LLO-treated cells received with increasing concentrations of CT extract showed fewer deaths in a dose-dependent manner (Figure 2E). Consistently, the live/dead cell staining showed that LLO incubation induced membrane-damaged cell death, which was reversed by CT extract administration. Taken together, the results showed that LDH release from damaged RAW264.7 cells was significantly reduced compared with the non-treated group when added to >32 μg/mL of CT extract (Figure 2F).

The most essential function of LLO has been considered to be the mediation of the intracellular survival of *L. monocytogenes*, which is critical to the pathogenesis of *L. monocytogenes* [32]. We examined the effects of CT extract on the intracellular replication of *L. monocytogenes* via an intracellular growth assay. As shown in Figure 2G, no significant changes were identified after 32/64 μg/mL of CT extract treatment for 0.5 h in an *L. monocytogenes* EGD infection system. However, the number of intracellular bacteria decreased after treatment for 3 h or 6 h when compared with no CT extract treatment. LLO, as a cytolysin, facilitated bacterial invasion and intracellular survival and induced host cell dysfunction [33]. Exposure to CT extract significantly attenuated LLO-mediated cell injury, suggesting that CT extract might prevent *L. monocytogenes* virulence via the inhibition of LLO potency, and this is congruent with increased survivals of macrophages after CT extract therapy, as evidenced by decreased bacterial loads.

### 2.3. Action Mechanism Assay

**Figure 3:**
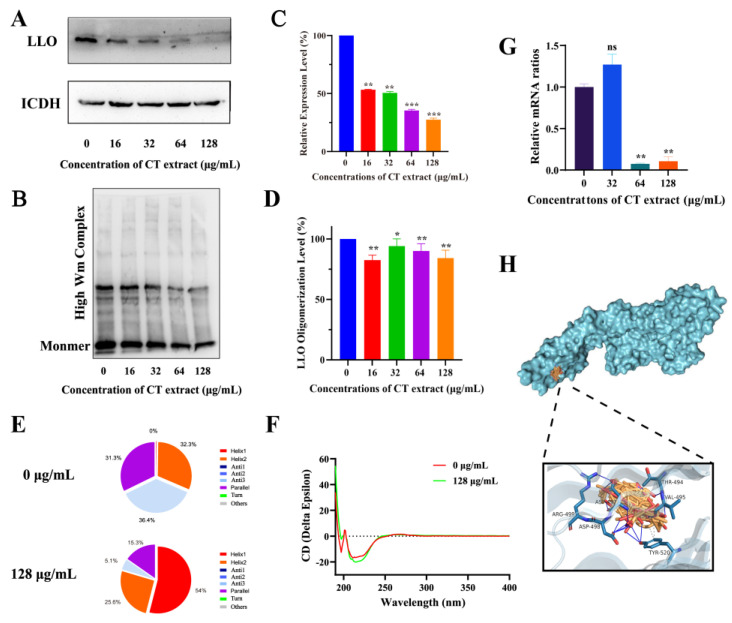
The CT extract inhibition on LLO expression and oligomerization. (**A**) Western blot analysis of the expression of LLO protein in culture supernatant. (**B**) Western blot images were used to observe the effect of CT extract on the oligomerization of LLO. Optical densities of the LLO expression (**C**) and the LLO oligomer level (**D**) were analyzed by ImageJ software. (**E**) Changes in LLO secondary structure with and without CT extraction. (**F**) Circular dichroism of LLO in the presence or absence of CT extract. The wavelength for CD spectroscopy was set as 190–250 nm. (**G**) CT extract inhibited the transcription of *hly* gene in *L. monocytogenes*. Bacteria were co-cultured with different concentrations of CT extract and the transcription of *hly* was evaluated by RT-PCR. (**H**) Molecular docking analysis was performed to predict the potential binding sites of LLO (4CDB) and ingredients of CT extract. The main components of CT extract binding with LLO pockets were viewed as a blue-grey surface. The ingredients were displayed in a stick model with carbon atoms in yellow and oxygen atoms in red. The binding site is mainly a hydrophilic binding pocket, and the hydrophilic residues were THR494, VAL495, TYR520, ASP497, ASP498 and ASP499. * *p* < 0.05, ** *p* < 0.01 and *** *p* < 0.001 compared to the positive group. ns, not significant.

LLO expression and oligomerization at the protein level were analyzed by Western blotting (WB) assays. The expression of LLO was inhibited with different concentrations of CT extract treatment (Figure 3A,C). LLO is initially secreted as a monomer, then promptly binds to the cell membrane. Immediately following cytolytic activity, oligomerization leads to the formation of pores and an outflow of cell contents [33]. The oligomerization of LLO was significantly decreased when treated with different concentrations of CT extract (Figure 3B,D). The results showed that CT extract significantly reduced the yield of LLO high molecular weight complexes, indicating that CT extract significantly inhibited LLO-induced hemolysis in vitro due to the inhibition of the oligomerization process of LLO. In addition, a significant change in the secondary structure of LLO was observed using the circular dichroism method, and the percentage of α-helix 2, anti 3 and parallel conformation in LLO decreased during treatment with CT extract; however, the percentage of α-helix1 conformation in LLO increased. The beta sheet twist of the secondary structure in the BeStSel method was very important and caused a strong effect on the CD spectrum. (Figure 3E,F). Upon further investigation, the transcript levels of the *hly* gene in *L. monocytogenes* EGD were not significantly different when treated with 32 µg/mL of CT extract, but a significant reduction was found when treated with 64/128 µg/mL (Figure 3G), which is in line with the results above. These results suggest that CT extract can inhibit the LLO in both the protein and gene levels.

The molecular docking of the LLO–CT extract complex was performed to investigate the molecular mechanisms of CT extract-induced pore formation inhibition. The main components of CT extract were screened using OB > 30% and collected by SailVina final v1.0 software to dock the CT extract with LLO; this phenomenon was then visualized in PyMOL as a graph. A lower free energy indicates a more stable binding, and the chemicals with lowest score bind completely in the activity pocket, as shown in Figure 3H. In particular, hydrogen bonds were formed between the top 6 donors and amino acid residues at the LLO active site, including THR494, VAL495, TYR520, ASP497, ASP498 and ASP499. These combined interactions formed by the interaction of CT extract at LLO active sites illustrated its attachment and the strength of interaction required for the inhibition efficacy of CT extract against the LLO pore-forming function, which is necessary for the inhibitory effect of CT extract on the LLO pore formation function. Taken together, our results indicated that CT extract, through multiple ingredients, can interfere with the structure of LLO and thus affect the activity of LLO. LLO punctures the cytomembrane via the assembly of monomers into oligomeric structures in a cholesterol- and time-dependent manner [34]. Consistently, our results established that CT extract efficiently blocked the pore-forming activity of LLO at the oligomerization stage, significantly ameliorating LLO-induced cytotoxicity as a result.

### 2.4. Network Pharmacology Analysis

**Figure 4:**
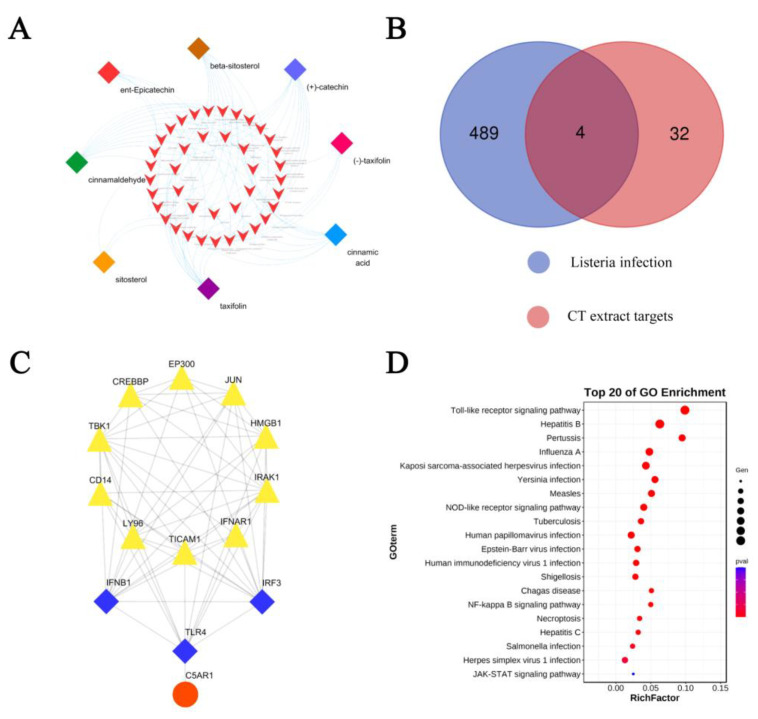
Functional enrichment analysis. (**A**) Ingredient-gene network of CT extract from TCMSP database with OB > 30%. DL > 0.18. (**B**) Venn diagram of the potential targets of CT extract and the therapeutic targets for *L. monocytogenes* disease. (**C**) Sub-network of overlapped genes related to neighbor genes was constructed using Cytoscape3.7.2. (**D**) GO enrichment analysis on overlapped genes and related neighbor genes.

**Figure 5:**
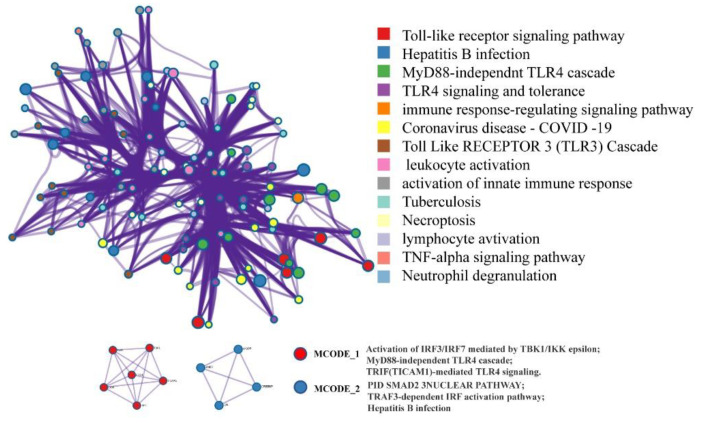
The PPI (Protein-Protein Interactions) network and MCODE classification of related genes in sub-network.

Network pharmacology has been widely used for drug discovery and development. To explore the potential pharmacological mechanisms of CT extract in the treatment of *L. monocytogenes* infection, we collected the target genes of the six main compounds in CT extract based on the TCMSP database (Figure 4A), and disease-related genes for listeria infection were obtained from the KEGG databases. Then, the common genes in both the six ingredients and the listeria infection disease-related genes were presented in a Venn diagram (Figure 4B). C5AR1, IFNB1, TLR4 and IRF3 were the results from the above analysis and then they further constructed the sub-network with the hprdPPI using the Cytoscape 3.6.0 (Figure 4C). The data were downloaded as bubble charts from the OmicShare database for the Gene Ontology (GO) analysis (Figure 4D). The main pathways were classified by MCODE_1 and MCODE_2 of the enriched terms network (Figure 5). All the results displayed above demonstrated that the toll-like receptor (TLRs) signaling pathway might be another potential target through which the components of CT combat the *L. monocytogenes* infection. TLRs are an important family of immune receptors discovered in recent years that recognize pathogens, immediately initiate innate immunity and initiate acquired immunity through signaling, which is necessary for the efficient elimination of invading pathogens [35]. TLR activation results in the production of inflammatory mediators, including cytokines, chemokines and interferons and TLRs are reported to be a promising, feasible drug target for antibacterial therapeutic strategies [36]. Some studies have shown that the inhibition of MAPK and NF-κB signals driven by small molecule compounds are not completely mediated by LLO, but the small molecules also effectively inhibit the myD88-dependent inflammatory response downstream of TLR 2/4 [37]. With the discovery of host recognition receptors and an increased understanding of innate immune signaling pathways, it has been shown that *L. monocytogenes* is also capable of avoiding detection through other mechanisms. These include the modification of bacterial ligands with pattern recognition receptors in innate immunity, modulation of host signaling pathways and targeting of host immune effector cells, thereby altering innate host defenses. This study suggests that CT extract may enhance resistance to *L. monocytogenes* by interacting with the host’s innate receptors.

### 2.5. Animal Experiments

**Figure 6:**
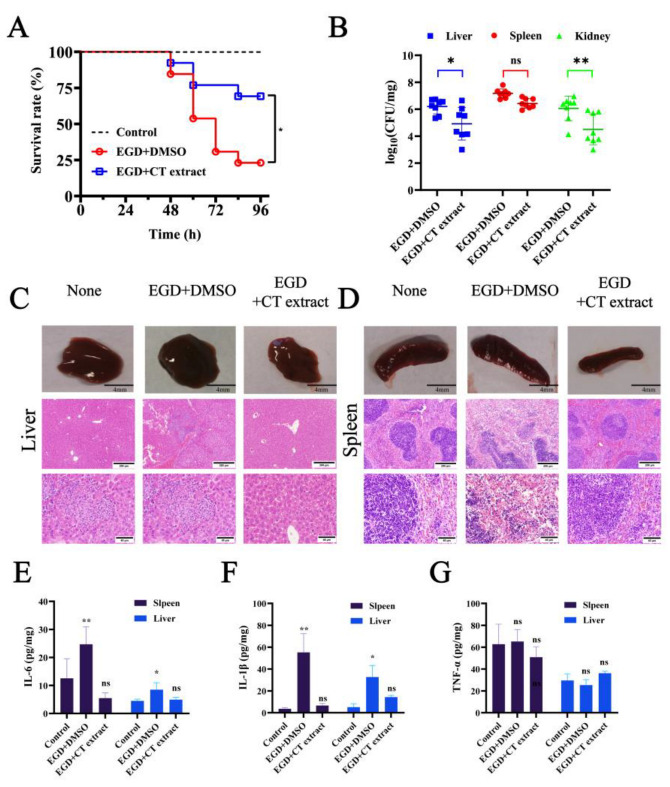
CT extract protects mice from *L. monocytogenes* infection. (**A**) Survival analysis of infected or uninfected mice treated with CT extract. Mice were infected with lethal doses of *L. monocytogenes* EGD and observed every 12 h. (**B**) Bacterial burden in liver and spleen determined within 48 h after infection in control (non-CT extract-treated) and CT extract-treated groups. Data are expressed as the means ± SEM. *p* values were calculated using one-tailed Mann–Whitney test (** *p* < 0.01). Histopathological analysis of livers (**C**) and spleens (**D**) from untreated mice infected with *L. monocytogenes* EGD with or without CT extract treatment was determined after 48 h infection. These images were collected from the representative stained sections, with the original magnification being 10 × (the scale length is 200μm) and 40 × (the scale length is 40μm), respectively. Cytokines of IL-6 (**E**), IL-1β (**F**) and TNF-α (**G**) in supernatants were examined using ELISAs. All the data are expressed as means ± S.D. (n ≥ 5). * *p* < 0.05 and ** *p* < 0.01.

Intraperitoneally *L. monocytogenes*-infected mouse models were used to evaluate the protective efficacy of CT extract. The results from the untreated group had an 80% mortality on day 4 of infection, and mortality in the 250 mg/kg CT extract-treated group was reduced to 25% on day 4 of infection (Figure 6A). The bacterial burden in the liver and kidney of CT extract-treated mice receiving sublethal doses of *L. monocytogenes* was significantly lower compared to controls at 48 h post-infection (Figure 6B). Histopathological analysis of the spleen and kidney was also performed to evaluate the treatment efficacy of CT extract. CT extract treatment led to a significant remission of pathological damage in the liver and spleen, as demonstrated by naked eye observation and histopathology. Liver cell necrosis, slight swelling and granular degeneration was observed in the untreated group in the pathological sections of the liver after being infected for 48 h (Figure 6C). The spleen of the mice in the untreated group showed a rapid enhancement in size, and it was blurred or shapeless around its edges (Figure 6D). In contrast, no obvious pathology was observed in the liver and spleen in the group treated with CT extract. The spleens of the CT extract-treated group were all similar to the control group. Indeed, *L. monocytogenes* manipulates the host’s cytolysis and inflammation response by a variety of mechanisms [13]. Subsequently, inflammatory factors (including IL-6 and IL-1β but not TNF-α) were significantly reduced in both the liver and spleen of the treated mice when compared with the infected group (Figure 6E-6G). These findings suggest that CT extract can interfere with and reduce the expression of inflammatory factors in mice, thus resisting *L. monocytogenes* infection.

### 2.6. Component Analysis

**Figure 7:**
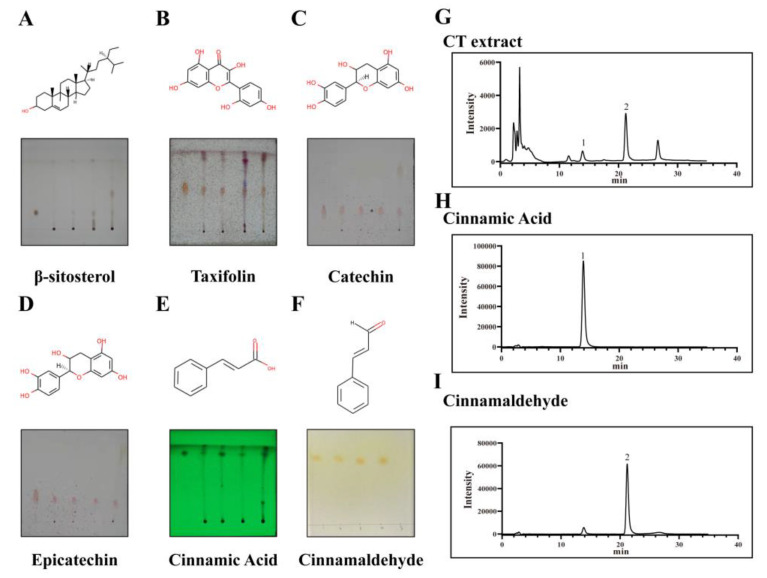
The ingredient identification of CT extract. TLC chromatogram and structural formula of β-sitosterol (**A**), taxifolin (**B**), catechin (**C**), epicatechin (**D**), cinnamic acid (**E**) and cinnamic aldehyde (**F**), respectively. HPLC chromatogram of CT extract (**G**), cinnamic acid (**H**) and cinnamic aldehyde (**I**). Peak 1 was the cinnamic acid response signal and peak 2 represented the cinnamic aldehyde response signal.

With 10 μL of the same concentration of CT extract (5 mg/mL) plated on TLC plates, β-sitosterol (Figure 7A), taxifolin (Figure 7B), catechin (Figure 7C) and epicatechin (Figure 7D) displayed obvious spots with unideal separation, whereas with 5 μL of CT extract at concentrations of 1 mg/mL plated on TLC plates, cinnamic acid (Figure 7E) and cinnamaldehyde (Figure 7F) exhibited good separation and obvious spots on the plates. The results of TLC demonstrated that CT extract had a higher content of cinnamic acid and cinnamaldehyde in contrast to other ingredients, and then the two components in CT extract (Figure 7G) were quantified by HPLC, with cinnamic acid (Figure 7H) displaying 0.44% ± 0.22% and cinnamaldehyde (Figure 7I) 0.76% ± 0.27%, respectively.

Natural plants have recently attracted greater attention around the world for their low cost, safety, reliability and long history of application, as well as for their extensive pharmacological potential to treat bacterial, viral and parasitic infections effectively [38]. Natural plant-derived multicomponent inhibitors have seldom been studied or reported. In this study, the hemolysis of both the *L. monocytogenes* supernatant-mediated and LLO protein-mediated could be significantly reduced after CT extract treatment, a multicomponent inhibitor. Meanwhile, CT extract can inhibit LLO expression at lower concentrations (Graphical Abstract). According to our results, CT extract efficiently inhibited the pore-forming activity of LLO at the oligomerization stage by directly binding to residues THR494, VAL495, TYR520, ASP497, ASP498 and ASP499. The inhibitory effect of CT extract on LLO also remains effective at the cellular level and may contribute to bacterial clearance using the host’s innate immunity. The best evidence of the therapeutic efficacy of CT extract is the significantly higher survival rates in the mouse infection model, which improved by 55% after treatment with CT extract. As modern medical research enters a new era combining science and technology with medical theory, cyber medical pharmacology aims to elucidate the interaction of active ingredients and targets of medicine on a molecular level [39,40]. In this study, network pharmacology and molecular docking approaches were used to predict the mechanism of CT extract in the treatment of *L. monocytogenes* infection and found that TLR signaling pathways might be another potential target through which the components of CT combat the *L. monocytogenes* infection [41]. Herein we provide a promising strategy for the development and utilization of herbal extracts.

## 3. Materials and Methods

### 3.1. Materials

#### 3.1.1. Microorganisms

*L. monocytogenes* EGD strains and *E. coli* BL21 (DE3) (pET21a-LLO) strains were used in this study, and EGD was supported by Dr. Masao Mitsuyama (Department of Microbiology, Kyoto University Graduate School of Medicine, Kyoto, Japan). The bacteria of *L. monocytogenes* were cultured with shaking at 37 °C in Tryptic Soy Broth (TSB; Qingdao Hope Biol-Technology Co., Ltd., Qingdao, China). Purified LLO was stored in our laboratory.

RAW 264.7 cells were cultured in Roswell Park Memorial Institute (RPMI) 1640 (Gibco, Waltham, MA, USA). The mouse mononuclear macrophages J774 and human osteosarcoma cells HeLa were cultured with Dulbecco’s modified Eagle’s medium (DMEM) (Gibco). Primary peritoneal macrophages were extracted from male C57BL/6 mice as previously described [22].

#### 3.1.2. Preparation of Plant Material

The OFL, CT, CC and BJC were obtained from ShaoHuaTang Chinese Medicine limited Co., Ltd. (AnHui, China). A voucher sample has been deposited in the herbarium of Jilin University.

The dried OFL, CT, CC and BJC were ground into powder. Subsequently, the powders were immersed and extracted twice with 10 volumes of 75% aqueous ethanol solution (*v*/*v*) at 70 °C for 2 h. The extraction liquids were filtered and mixed. Then, purified water was added at a ratio of 1:500, and the solution was incubated overnight at 4 °C. Then, the liquid was filtered to obtain the precipitate and evaporated in a vacuum to produce the dried extraction powder. The stock solutions (1 mg/mL) of the respective extract powder above were prepared by dissolving the powder with DMSO for further study. The CT bioactive ingredients, cinnamaldehyde and cinnamic acid, were identified using HPLC.

The standard substances (catechin, cinnamaldehyde, β-sitosterol, cinnamic acid, taxifolin and epicatechin) (≥98% purity) were provided by Chengdu Herbpurify Co., Ltd., Chengdu, China and solubilized to a storage density of 10,240 μg/mL with DMSO (Sigma-Aldrich, St. Louis, MO, USA).

### 3.2. Minimal Inhibitory Concentration (MIC) Assay

In accordance with the Clinical and Laboratory Standards Institute (CLSI) guidelines, the MIC assays of CT extract for *L. monocytogenes* were performed using the checkerboard microdilution method [42].

### 3.3. Growth Curve Assay

Growth curves were determined by previous reports [26]. The bacterial suspension was divided evenly into five flasks with different concentrations of CT extract (0, 16, 32, 64 and 128 μg/mL) and only supplemented with DMSO as a control.

### 3.4. Hemolysis Assay

Hemolysis assays were determined by previous reports [43]. The sample treated with DMSO was regarded as a negative control, and the sample treated with 0.2% Triton X-100 served as a positive control (100% hemolysis). The hemolysis was defined as the ratio of the OD570 value of each sample relative to the positive control.

In addition, purified LLO protein or untreated EGD culture supernatant incubated with different concentrations of CT extract was also used for hemolysis assay.

### 3.5. Cytotoxicity Analysis

The cytotoxicity of CT extract was evaluated using the LDH Cytotoxicity Test Kit (Roche, Penzberg, Germany) as previously described [44]. In brief, the cell culture supernatant was replaced with 200 µL of medium containing different concentrations of CT extract and the cells continued to incubate for 6 h under the same conditions. Moreover, samples treated with 0.2% Triton X-100 or RPMI-1640 only were set as the positive control and negative control, respectively. Next, the LDH in the culture supernatant was detected with a Cytotoxicity Detection Kit (LDH; Roche, Basel, Switzerland) and the final percentage of dead cells was shown as (OD_492 nm_ of sample-OD_492 nm_ of negative control)/(OD_492 nm_ of positive control-OD_492 nm_ of negative control) × 100%. The cell culture supernatants were measured using a microplate reader (Tecan, Austria) at 492 nm.

### 3.6. Intracellular Growth Assay

The RAW264.7 cells were used for the intracellular growth assay according to previous reports [25]. Briefly, the cells were infected with *L. monocytogenes* EGD at the multiplicity of infection. The resulting suspensions were inoculated onto TSB agar plates at 37 °C for 24 h. The colony-forming unit (CFU) value of intracellular bacteria at different time points was used to determine the inhibition efficacy of CT extract against *L. monocytogenes* invasion.

### 3.7. Cell Viability Determination

Purified LLO (0.5 μM) was incubated with different concentrations of CT extract for 30 min at 37 °C, and then the mixture was added to the well of 96-well plates to incubate with the cells for 5 h. The cells treated with DMEM and 0.2% Triton X-100 were used as the negative control and the positive control, respectively. The LDH detection method was the same as described previously. In addition, the treated cells were stained with live/dead reagent (Invitrogen, Carlsbad, CA, USA) and then photographed with a confocal laser scanning microscope (Olympus, Tokyo, Japan) [20].

### 3.8. Western Blotting Analysis

The expression level of LLO in EGD was evaluated through WB after treatment with different concentrations of CT extract (0, 16, 32, 64 and 128 μg/mL) for 6 h based on incubation at 37 °C. Subsequently, the aliquots of each bacterial suspension were centrifuged at 12,000 rpm for 10 min and processed for the SDS-PAGE assay in accordance with a previous report [45]. Then, the level of LLO was examined using LLO antibodies, as described in our previous study [25].

LLO was pre-incubated with or without the indicated concentrations of CT extract at 37 °C for 20 min and LLO oligomerization was induced in vitro, as previously described [46].

### 3.9. Circular Dichroism (CD) Analysis

CD analysis was conducted using a MOS-500 spectrophotometer (Bio-Logic, Seyssinet-Pariset, France) to test the secondary structures of LLO treated with or without CT extract (128 μg/mL) [21]. The secondary structural changes (e.g., α-helix, beta sheet and beta turnover of LLO protein) were investigated using a BeStSel Web server [47]. All samples achieved normalized root mean square deviation values lower than 0.1.

### 3.10. Real-Time Reverse Transcription Polymerase Chain Reaction (RT-PCR)

The *L. monocytogenes* EGD strain was cultured overnight, diluted with TSB medium and co-cultured with different concentrations of CT extract for 6 h. The total RNA was extracted in accordance with the manufacturer’s protocol using the RNeasy kit (TianGen, Sichuan, China) as previously described [48]. The primer pairs employed for RT-PCR primers were as follows: *hly*, 5′-GAGCCTAACCTATCCAG-3′ (forward) and 5′-TGTAAGCCATTTCGTCA-3′ (reverse); and 16S RNA, 5′-CTTCCGCAATGGACGAAAGT-3′ (forward) and 5′-ACGATCCGAAAACCTTCT-TCA T C-3′ (reverse) [20].

### 3.11. Molecular Docking

The crystal structure of LLO protein (4CDB) originated from the Research Collaboratory for Structural Bioinformation (RCSB) Protein Data Bank (PDB) (https://www.rcsb.org/) (accessed on 18 May 2022). The 2D structures of the main ingredients of CT extract originated from PubChem (https://pubchem.ncbi.nlm.nih.gov/) (accessed on 18 May 2022). In Sail Vina v1.0, the molecular docking process was performed and the results were imported into the protein–ligand interaction profiler (PLIP) website for analysis (https://plip-tool.biotec.tu-dresden.de/plip-web/plip/index) (accessed on 18 May 2022). The visual images were generated with PyMol v2.4.0 software [49].

### 3.12. Animal Experiments

Six- to eight-week-old female Balb/c mice weighing 20 ± 2 g were provided by Changsheng Biotechnology Co. Ltd. (Changchun, China). All animal experiments were performed in accordance with the guidelines of the Animal Care and Use Committee (ACUC) of Jilin University.

In the survival rate study, 1.5 × 10^7^ CFU of *L. monocytogenes* EGD was injected intraperitoneally into each mouse. After the mice had been infected for 2 h, the mice in the CT-extract-treated group were injected subcutaneously with 250 mg/kg CT extract, and the survival rates of different groups were recorded as (the number of mice alive/total number of experimental mice) × 100%.

The sublethal dose of *L. monocytogenes* EGD in 5 × 10^6^ CFU was injected intraperitoneally for the bacterial loading and pathological analysis of the target organs. The liver and spleen were fixed in 1% formalin and then stained with hematoxylin and eosin (H&E) to observe the injury under a light microscope (SANYO, Osaka, Japan). The *L. monocytogenes* EGD in the spleen and liver were cracked in 2% Triton X-100, diluted and inoculated onto TSB agar plates at 37 °C for 24 h to determine the number of colonies. The levels of cytokines (IL-1β, IL-6, TNF-α and IFN-γ) in the supernatants of homogenized spleen and liver tissue were detected using enzyme-linked immunosorbent assay (ELISA) [50].

### 3.13. The Detection of the Medicinal Ingredients of Cinnamon Twig and Network Pharma-Cology Analysis

The ingredients of the extraction above were identified using TLC and HPLC [51]. The methods are elucidated in the Supplementary Materials. The ingredient targets of CT extract originated from the PubChem database (https://pubchem.ncbi.nlm.nih.gov/) (accessed on 7 March 2022). The genes related to *L. monocytogenes* infectious disease were obtained from the Kyoto Encyclopedia of Genes and Genomes (KEGG) database (Kyoto Encyclopedia of Genes and Genomes, https://www.genome.jp/kegg/mapper/color.html) (accessed on 7 March 2022). Subsequently, the ingredient–target–disease–gene relationship was shown by Cytoscape 3.7.2 and the Venn diagram presented common genes in both databases [52]. The common gene-related subnetworks associated with Human Protein Reference Database Protein–Protein Interactions (hprdPPI) were further mined in the Cytoscape software. The related pathways of interacting genes were enriched by STRING or Metascape databases (https://cn.string-db.org/, http://metascape.org/) (accessed on 7 March 2022) and a bubble diagram of the KEGG assay was generated from OmicShare online (http://www.omicshare.com) (accessed on 7 March 2022).

### 3.14. Statistical Analysis

GraphPad Prism 8.0 was applied for the statistical analysis. All experimental data were analyzed through the Student’s *t*-test and represented as the means ± standard deviation (SD), *p* < 0.05 (*) and *p* < 0.01 (**), showing that the difference achieved statistical significance.

## 4. Conclusions

In summary, the inhibitory effect of CT extracts on LLO provides a new treatment measure for the use of natural herbal compounds as an alternative therapy for *L. monocytogenes* infection. Our study confirms that CT extracts, containing a variety of complex chemical components, can act as effective inhibitors of LLO formation and have good therapeutic effects on *L. monocytogenes* infections in vivo. CT extracts can provide new ideas and methods for the development of antibacterial infection strategies. Due to its characteristic ability to inhibit bacterial virulence, CT extract is a promising candidate drug for the treatment of *L. monocytogenes* infection.

## Data Availability

The data in this study are available in this article.

## Supplementary Materials

The following supporting information can be downloaded at: https://www.mdpi.com/article/10.3390/molecules28041625/s1.

## Abbreviations

ActA, actin aggregation protein; BJC, bark of Japanese cinnamon; CC, *Cinnamomi cortex*; CD, circular dichroism; CDC, cholesterol-dependent cytolysin; CFU, colony-forming unit; CT, cinnamon twig; DMEM, Dulbecco’s modified Eagle’s medium; ELISA, enzyme-linked immunosorbent assay; ER, endoplasmic reticulum; OFL, *Osmanthus fragrans Lour*; HPLC, high-performance liquid chromatography; KEGG, Kyoto Encyclopedia of Genes and Genomes; LDH, lactate dehydrogenase; *L. monocytogenes, Listeria monocytogenes;* LLO, Listeriolysin O; MIC, minimal inhibitory concentration; MOI, multiplicity of infection; RPMI, Roswell Park Memorial Institute; RT-PCR, reverse transcription-polymerase chain reaction; TLC, thin layer chromatography; TLRs, toll-like receptors; TSB, TSB Medium; WB, Western blotting.
