# Supplementary material for: The Application of Cinnamon Twig Extract as an Inhibitor of Listeriolysin O against Listeria monocytogenes Infection
Source: Molecules. 2023 Feb 8;28(4):1625. doi: 10.3390/molecules28041625 (PMC9962927; doi:10.3390/molecules28041625)
Supplement: Supplementary file 1 [file molecules-28-01625-s001.zip › Supplemental File.docx]

**Thin-layer chromatography (TLC) and high-performance liquid chromatography (HPLC) methods of the main ingredients of *Cinnamon* Twig (CT) extract**

**CT extract stock solution 1:** CT extract was added into the anhydrous ethanol to final concentration of 5mg/mL, sonicate for 10 min, centrifuge (8000r/min, 10min), taked 10 μL of supernatant and spoted it on a silica gel G thin-layer plate.

**CT extract stock solution 2:** The supernatant of CT extract stock solution 1 was diluted 5 folds with anhydrous ethanol to final concentration of 1mg/mL.

**Identification of β-sitosterol by TLC**

β-sitosterol reference substance were added into anhydrous ethanol to prepare reference substance solutions (1mg/mL). CT extract stock solution 1 and reference standard solution were drawed 10μL each placed on the same silica gel G thin layer plate, the stock solution was repeated four times in parallel. Then unfolded with cyclohexane-ether-ethyl acetate (20: 5.5: 2.5), and drying, spraying with 10% sulfuric acid ethanol solution, and heating until the spots showed clear color. Spots of the same color appear in the test chromatogram at the locations corresponding to the chromatograms of the reference substances.

**TLC identification of catechin and epicatechin**

Catechin reference substance and epicatechin reference substance were added with methanol to prepare a mixed solution containing 0.2mg per 1ml, which was used as the reference substance solution. CT extract stock solution 1 and reference standard solution were sucked 10μL each and applied to the same cellulose prefabricated plate respectively, and n-butanol–acetic acid-water (3: 2: 1) was used as the developing agent to unfold, take out, dry in the air, spray with 10% sulfuric acid ethanol solution, and heat until the spots showed clear color. The test article chromatogram shows the same red spot at the location corresponding to the reference article chromatogram.

**TLC identification of taxifolin**

A separate solution of 1mg per 1ml of taxifolin reference standard in methanol was used as the reference standard solution. CT extract stock solution 1 and reference standard solution were sucked10μL each, placed on the same silica gel G thin layer plate respectively, and expanded with petroleum ether (60–90 ℃)–ethyl acetate–formic acid (10:11:0.5) as the developing agent, taken out, dried, sprayed with 10% sulfuric acid ethanol solution, and heated at 105℃ until the spots showed clear color. Spots of the same color appear in the test chromatogram at locations corresponding to the chromatograms of the reference substances.

**TLC identification of cinnamic acid**

A separate solution of 1mg per 1ml of cinnamic acid reference standard in ether was used as the reference standard solution. CT extract stock solution 2 and reference standard solution were aspirated 5μL each and placed on the same silica gel GF254 TLC plate, respectively, at 10–15℃ with petroleum ether (30–60℃)–n-hexane–ethyl formate–formic acid (10: 30: 15: 1) as the developing agent, and taken out, dried and examined under an ultraviolet lamp (254nm). Spots of the same color appear in the test chromatogram at locations corresponding to the chromatograms of the reference substances.

**TLC identification of cinnamic aldehyde**

Cinnamic aldehyde reference substance was added with ethanol to prepare a solution containing 1mg per 1mL, which was used as the reference substance solution. CT extract stock solution 2 and reference standard solution were sucked 5μL each, placed on the same silica gel G thin layer plate respectively, and expanded with petroleum ether (60–90℃)-ethyl acetate (17:3) as the developing agent, taken out, dried, and sprayed with dinitrophenylhydrazine ethanol test solution. Spots of the same color appear in the test chromatogram at locations corresponding to the chromatograms of the reference substances.

**HPLC method of the main ingredients**

Prepare 1mg per 1mL of cinnamic aldehyde and cinnamic acid reference standard in methanol as the reference standard solution. According to the determination of HPLC method in Chinese Pharmacopoeia Commission, 2020, the right amount of test solution of CT extract stock solution 2 and reference solution were injected into HPLC. A C18 chromatographic column (Shimadzu, Japan,250 mm×4.6 mm,5 μm) was used and temperature was maintained at 35 °C. Solvents A (0.1% formic acid) and B (acetonitrile) were used as mobile phases at a flow rate of 1 ml/min. A gradient condition of 0 min, 90% A; 12 min, 80% A; 35 min, 50% A; 40 min, 0% A; 42 min, 90% A was used throughout the run. Chromatograms were acquired at wavelengths 265 nm.
